# Ion Current Sensor for Gas Turbine Condition Dynamical Monitoring: Modeling and Characterization

**DOI:** 10.3390/s21206944

**Published:** 2021-10-19

**Authors:** Tommaso Addabbo, Ada Fort, Elia Landi, Marco Mugnaini, Lorenzo Parri, Valerio Vignoli, Alessandro Zucca, Christian Romano

**Affiliations:** 1 Department of Information Engineering and Mathematics, University of Siena, 53100 Siena, Italy; addabbo@dii.unisi.it (T.A.); elialandi@gmail.com (E.L.); parri@diism.unisi.it (L.P.); mugnaini@diism.unisi.it (M.M.); vignoli@dii.unisi.it (V.V.); 2 Baker Hughes, 50127 Florence, Italy; alessandro.zucca@bakerhughes.com (A.Z.); Christian.Romano@bakerhughes.com (C.R.)

**Keywords:** condition monitoring, ion current sensing, gas turbines

## Abstract

This paper aims to thoroughly investigate the potential of ion current measurements in the context of combustion process monitoring in gas turbines. The study is targeted at characterizing the dynamic behavior of a typical ion-current measurement system based on a spark-plug. Starting from the preliminary study published in a previous work, the authors propose a refined model of the electrode (spark plug), based on the Langmuir probe theory, that incorporates the physical surface effects and proposes an optimized design of the conditioning electronics, which exploits a low frequency AC square wave biasing of the electrodes and allows for compensating some relevant parasitic effects. The authors present experimental results obtained in the laboratory, which allow for the evaluation of the validity of the model and the interpreting of the characteristics of the measurement signal. Finally, measurements carried out in the field on an industrial combustor are presented. The results confirm that the charged chemical species density sensed by the proposed measurement system and related to the mean value of the output signal is an indicator of the ‘average’ combustion process conditions in terms e.g., of air/fuel ratio, whereas the high frequency spectral component of the measured signal can give information related to the turbulent regime and to the presence of pressure pulsations. Results obtained with a prototype system demonstrated an achievable resolution of about 5 Pa on the estimated amplitude, even under small biasing voltage (22.5 V) and an estimated bandwidth of 10 kHz.

## 1. Introduction

An important part of turbomachine condition monitoring lies in combustion monitoring. Currently, reducing the emissions is a key issue in all combustion devices, such as gas turbines. This has led to new combustor techniques and designs, the most important being the lean premixed combustor, where the mixing of air and fuel leads to a lean combustion whose effect is to reduce the flame temperature, and thus the NO_x_ emissions [1]. This kind of combustor is now applied to land based heavy duty gas turbines for mechanical drives in heavy industry or power generation, and in turbines for aircraft applications. However, by operating very lean to reach low NO_x_ and particulate matter emission, the combustors have limited margins among stable combustion, lean-flame instabilities and lean blowout. The lean combustion is mainly critical when applied in engines operating in dynamic regimes, such as in aircrafts, due to the wide range of working points; here the main risk is the lean blowout (LBO), especially in transient operations given by rapid throttle variations. On the other hand, blowouts are also an expensive problem in industrial system powertrains where a blowout can lead to significant economic losses [2].

The signatures of a blowout condition can be recognized in localized extinction and reignition events and irregular rates of fuel consumption. In more detail, unstable combustion is related to self-sustained combustion oscillations at or near the acoustic frequency of the combustion chamber, which are the result of the closed loop coupling between unsteady heat release and pressure fluctuations [3,4,5].

Currently, many blowout monitoring approaches are based on sensing the low-frequency combustion oscillations that precede the blowout event. Usually, standard high-temperature pressure-transducers are used for this application [6,7], together with optical measurement systems which exploit the chemiluminescence principle given by the electronically excited molecules generated by fuel oxidation [8,9].

In this work, the focus is to enhance the reliability of the combustion monitoring system by exploiting pre-existing components embedded in the combustion chamber, thereby avoiding critical modification to the turbomachine structure. The final aim is improving the capability of monitoring stable and unstable combustion conditions.

In particular, this paper investigates the possibility of integrating the information gathered by traditional pulsation monitoring, obtained with pressure sensors, with those obtained from an ion-sensor realized exploiting the electrode of the built-in spare spark-plug. This added sensor can provide redundancy to the monitoring system, without requiring further hardware modifications of the machinery’s mechanical structure. Moreover, the additional ion-sensor is obtained from a pre-existing rugged component, therefore it can significantly increase the reliability of the measurement setup. Even if this approach was already proposed in the literature [10,11] (and the references therein), a complete modeling of the sensor and of the measuring system specifically aimed at understanding the measurement performance, especially devoted to dynamic measurements, is still lacking.

It is worth noting that the most recent approaches to combustion and machine monitoring rely on artificial intelligence (AI) for decision support, and significant results have been reached by applying this approach to gas turbine monitoring [12,13,14,15,16]. However, the success of AI, and more generally of any data processing technique, relies on the availability of reliable and accurate sensor data as input. Indeed, sensor performance can determine the success of the condition monitoring system and the degree of improvement in machine performance [17] to the extent that system fault detections may sometimes be due to multiple on-board sensor failures. In this sense, the development of reliable and accurate sensing techniques and devices and the analysis of the effect of new application environments for known sensing strategies is of utmost importance as ancillary activities to subsequent data processing and interpretation. Additionally, highlighting the type and amount of information buried in the sensor signals facilitates the task of extracting meaningful features from the raw data for subsequent high-level data processing.

Ion sensing is based on immersing one or two metal electrodes in the flame where exothermic chemical reactions release a large amount of ions (chemo-ionization) and, sensing their concentration, either biasing the electrodes and measuring the current due to the drift of ions forced by the external applied electrical field, or by using unbiased electrodes and by measuring the charge induced in the electrodes by the ions/electrons freely moving in front of the electrodes themselves [18,19,20,21,22,23]. The signal thus obtained depends on the ion concentration in the flame and allows for monitoring many combustion process characteristics, among which pressure pulsations that affect the ion concentration locally, in addition to simply indicating the presence of the flame.

In general, many different monitoring applications through ion sensors have been proposed for combustors or combustion engines, mainly for endothermic reciprocating engines, and more rarely for rotary ones [10]. The simplest successful application is flame detection, but recently many studies have proposed systems with enhanced monitoring capabilities, from crank angle in diesel engines to burning gas characteristics in gas turbines. In these studies, some efforts were devoted to understanding and modeling the sensor behavior, but a thorough model still lacks. On the other hand, the same sensor structure and sensing strategy is used, studied and modeled for plasma studies in different application fields (plasma-based material processing and aerospace application) and it is known as a Langmuir probe. In this paper, based on this theory, a complete model for the ion sensor is developed and compared with the simpler ion sensor models used in the literature. The model refines previous studies [10] by also considering the physical effect of the gas/electrode interfaces. Note that chemical or electrochemical effects at this boundary are not considered. The model also describes the loading effect of the front-end circuit and the overall dynamic behavior. The paper is organized in the following way. In Section 2, the sensor model is presented. Section 3 describes the experimental set-up. In Section 4, the model is verified through laboratory tests and preliminary results concerning in-field testing are reported.

## 2. Measurement System Model

### 2.1. Sensor Model

**Figure 1:**
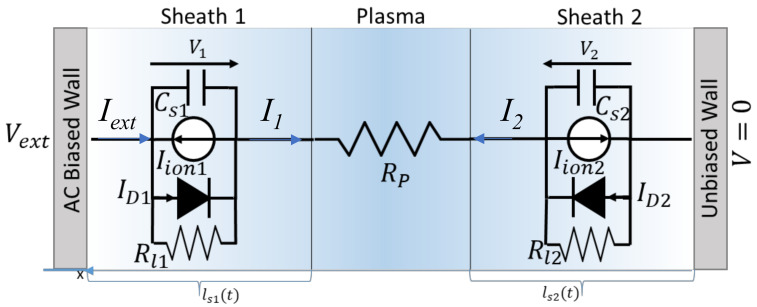
Equivalent circuit of a plasma between two walls.

In this section the equivalent circuit for the ion current sensor, shown in Figure 1, is presented. This model is based on the classical Langmuir probe, assuming that the burning gas behaves as a plasma [21,24], and it accounts for the electric transport in the plasma and for the physical (not chemical) interaction between the plasma and the electrodes [22,23]. In the case of interest, one electrode is the spark plug tip whereas the other one is the spark plug casing.

The circuit in Figure 1 describes the two electrodes as conductive walls (i.e., provides a 1-D model), and accounts for the presence of a charge space region, the Debye sheath at each electrode/plasma interface through the parallel of a diode, a current generator, a resistance and a capacitance.

In detail, the model consists of the series of two non-interacting solid/plasma interfaces corresponding to the two electrodes, and of a resistance, *R_p_,* representing the gas path between the two electrodes. This description assumes that the two electrodes are sufficiently far, i.e., that L, the effective distance between the electrodes, is much larger than the Debye length, therefore the central region far from the walls can be assumed to behave as bulk plasma.

The equivalent circuit of each of the two electrode/plasma interfaces is found by exploiting the relationship between the transport current $I_{t}$ flowing across an electrode with area *A*, at a voltage *V* in contact with a plasma at the potential $V_{p}$, with *V* < *V_p_*, that can be written as follows:

(1) $$I_{t}=en_{s}Av_{b}^{\prime }\sqrt{1+\frac{2e\left(V_{p}-V\right)}{kT_{e}}}-en_{s}Av_{e}^{\prime }-en_{s}Av_{e}^{\prime }\left(e^{\frac{-e\left(V_{p}-V\right)}{kT_{e}}}-1\right)$$

where *k* is Boltzmann constant, *e* is the electron charge, *T_e_* is the electron temperature, $v_{e}^{\prime }$ is the average speed of the electrons that can reach the electrode, overcoming the potential barrier in the sheath, and $v_{e}^{\prime }=\sqrt{\frac{kT_{e}}{2\pi m_{e}}},$ being *m_e_* the electron mass. $n_{s}$ is the density of electrons at the edge of the sheath far from the electrode and we have $n_{s}≅0.61n_{b}$, with *n_b_* the bulk density of electrons in the plasma. Finally, $v_{b}^{\prime }=e^{\frac{1}{2}}v_{b}$, being $v_{b}=\sqrt{\frac{kT_{e}}{m_{i}}}$ the Bohm velocity of ions.

The expression of the current reported in Equation (1) is found by considering the typical working conditions in the combustion chamber [21,24,25], as described in Appendix A, where a detailed derivation of Equation (1) is provided. 

The first term of the current in Equation (1) is related to the ion transport and it is found exploiting the energy conservation for ions traveling in the sheath, therefore accounting for the accelerating effect of the electric field on ions. This term can be linearized, using a first order Taylor expansion, around a selected working point *V_p_* − *V* = *V*_*W*_, as follows:

(2) $$I_{si}=eAn_{s}v_{b}^{\prime }\sqrt{1+\frac{2e\left(V_{p}-V\right)}{kT_{e}}}\approx eAn_{s}v_{b}^{\prime \prime }+\frac{1}{R_{l}}\left(V_{p}-V\right)$$

where $R_{l}={\left.{\left(\frac{dI_{si}}{d\left(V_{p}-V\right)}\right)}^{-1}\right|}_{V_{p}-V=V_{W}},$ and $v_{b}^{\prime \prime }=v_{b}^{\prime }\;\left[\sqrt{1+\frac{2eV_{w}}{kT_{e}}}-\frac{\frac{e}{kT_{e}}V_{w}}{\sqrt{1+\frac{2eV_{w}}{kT_{e}}}}\right]$.

Under this approximation Equation (1) can be rewritten as:

(3) $$I_{t}=\frac{1}{R_{l}}\left(V_{p}-V\right)+en_{s}A\left(v_{b}^{\prime \prime }-v_{e}^{\prime }\right)-en_{s}Av_{e}^{\prime }\left(e^{\frac{-e\left(V_{p}-V\right)}{kT_{e}}}-1\right)$$

and can be represented using the parallel of a resistance, a current generator and a diode under a voltage *V*. $I_{t}$ represents the transport current across the electrode/plasma interface, on the other hand the displacement current $I_{C}$ across this interface caused by the variation of the sheath charge $Q_{s}$ over time can be written as follows:

(4) $$I_{C}=\frac{dQ_{s}}{dt}=\frac{dQ_{s}}{d\left(V_{p}-V\right)}\frac{d\left(V_{p}-V\right)}{dt}=C_{S}\frac{d\left(V_{p}-V\right)}{dt}$$

where $C_{S}$ is the sheath capacitance related to the sheath thickness, $l_{s},$ and to the wall area A, as:

(5) $$C_{S}=\frac{\epsilon A}{l_{s}}$$

and where *l_s_* can be assumed approximately equal to the Debye length.

Concluding the total current flowing across the electrode, *I*, can be found as the superimposition of $I_{c}$ and $I_{t}$, such that $I=I_{t}+I_{c}$.

The expression of the current in Equations (3) and (4) can be used to obtain the equivalent circuit reported in Figure 1, with the values of components specified in Table 1. 

Note that if $V>V_{p}$, the role of electrons and ions is switched, and the model must be changed accordingly, as specified in Table 1.

All the circuit parameters, reported in Table 1, depend on the electrode areas, $A_{j}\;\left(j=1,2\right)$. Note that in the considered case only one electrode is driven, whereas the other one is grounded, therefore the referenced electrode is virtually the whole metal surface enclosing the plasma, i.e., the whole combustor surface. Therefore, we have *A*_2_ ≫ *A*_1_.

To complete the sensor model, with reference to Figure 1, the resistance *R_p_*, represents the transport current in the bulk plasma and can be evaluated by [26,27]:

(6) $$R_{p}=\frac{0.61L}{n_{s}A_{1}e\;\mu_{e}}$$

where $\mu_{e}$ is the electron mobility in plasma. Equation (6) is found by neglecting the contribution of ions, since the ion density and the electron density are equal in the bulk plasma, whereas their mobilities are very different ($\mu_{e}\gg \mu_{i}$). The flame plasma between the electrodes can be usually considered as a good conductor and $R_{p}$. a small resistance. 

Using the parameters in Table 1, and Equation (6) the static I-V characteristic of the ion sensor can be numerically evaluated, by solving the following equations:

(7) $$I_{ext}=I_{1}=-I_{2}$$

(8) $$V_{ext}=V_{1}-V_{2}+R_{p}I_{ext}$$

**Figure 2:**
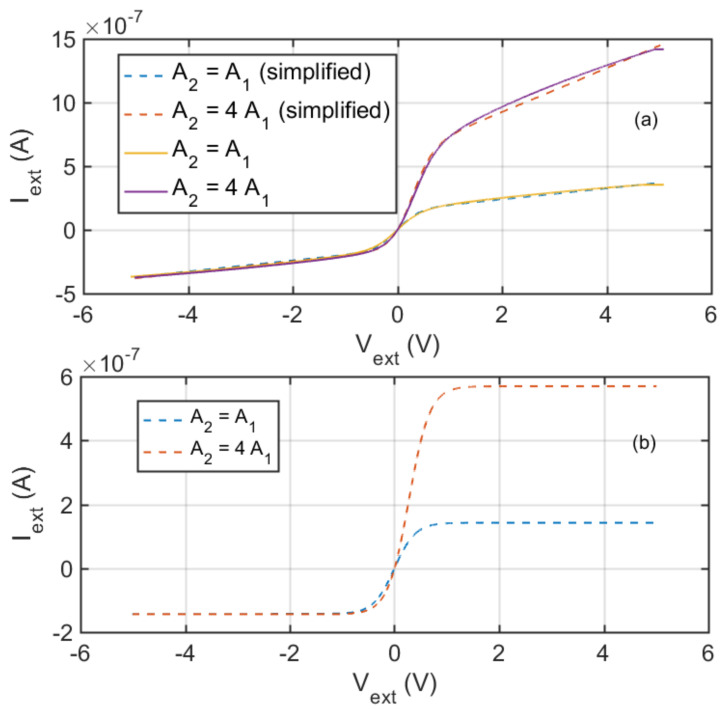
Static I-V characteristics. (**a**): Results obtained applying the model in Equation (1) (solid lines) and the simplified model of Equation (3) and Figure 1 (dashed lines) with the circuit parameters defined in Table 1. (**b**): Results obtained applying the simplified model of Equation (3) and Figure 1, without the resistances.

As an example of the results obtained with the proposed model, Figure 2a shows the I−V static characteristics of an ion sensors characterized by two different ratios between the electrode areas, with $A_{2}\geq A_{1}$, but assuming *v*_*e*1_′*A*_1_ > *v*_*b*2_′*A*_2_. The displayed data are obtained with *A*_1_ = 7 × 10^−6^ m^2^, *n_s_* = 4.3 × 10^13^ m^−3^, *m_i_* = 19 Da (H_3_O^+^), *μ_e_* = 800 m^2^/(Vs), *T_e_* = 2273 K. It can be observed that the asymmetry of the electrode geometry implies a behaviour which can be defined as ‘rectifying’, with an asymmetric shape for positive or negative sensor biasing. Note that if in the equivalent circuit, the two resistances are not considered (which corresponds to considering a constant speed of the ions/electrons in the sheath when *V* < *V_p_*/*V* > *V_p_*) the current reaches a saturation value both for positive and negative biasing due to the ion current contributions, as shown in Figure 2b with magnitudes for the saturation currents:

(9) $$\left|i_{p1.2}\right|\approx eA_{1,2}n_{s}v_{b}^{\prime }$$

The rectifying behaviour in the considered cases derives from the different values of the two ion currents due to the different values of *A_j_*. On the contrary, if *v*_*e*1_′*A*_1_ < *v*_*b*2_′*A*_2_ then the saturation current, $i_{p_{2}}$ is due to electrons and $\left|i_{p_{2}}\right|\approx eA_{1}n_{s}v_{e}^{\prime }$.

From the data reported in Figure 2a, it can be seen that the linear approximation of the *I_si_* term provides very good results. Moreover, when biasing the sensor with a large positive voltage, *V_W_* and operating dynamically around this working point, the sensor can be satisfactorily approximated by the resistance with value *R*_l2_ with a parallel capacitance *C*_*s*2_.

Considering the most relevant features of the I-V sensor characteristics, in this paper we propose a simplified model exploiting two ‘true diodes’ which are described by the right term of Equation (3) both for *V_j_* > 0 and for *V_j_* < 0, i.e., exploiting only the relationship in the first row of Table 1 (neglecting the effect of switched role between ions and electrons in case of *V_j_* < 0), adapting the constant current generator value, and using a constant resistance with the value defined in Equation (2), i.e., assuming a linear relationship between the external voltage and ion current magnitude. This circuit can be easily used to simulate the behavior of ion sensors exploiting electrical circuit simulators as, for instance, Spice.

### 2.2. Response of the Ion Sensor to Small Dynamic Signals

**Figure 3:**
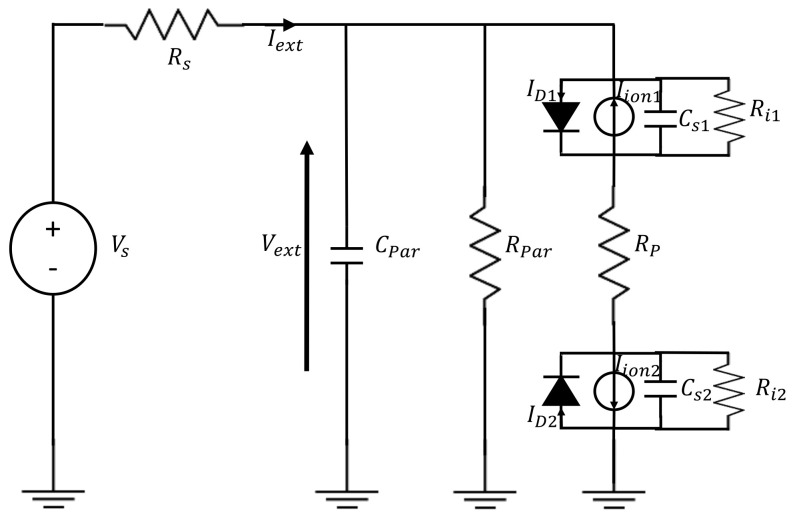
Measurement setup equivalent circuit. *C_Par_* and *R_Par_* model the cable parasitic capacitances and resistive losses.

The transfer function of the measurement system based on the ion current sensor in the frequency domain was derived considering a large positive biasing, the spark plug linearized equivalent circuit, the main parasitic components, the cable equivalent circuit, and the equivalent circuit of the front-end electronics as reported in Figure 3. The front-end electronics is represented by a voltage generator in series with a shunt resistance used for current sensing. In details, the resistance *R_s_* acts as the shunt resistor (I-V converter) of an amperometric circuit. 

In general, it can be shown that even if the parasitic sheath capacitances $C_{s1}$ and $C_{s2}$, determined using Equation (5), vary with the electrodes biasing, because they depend on the sheath thickness, their value also in the worst-case scenario is much smaller than the parasitic capacitance of the cable, $C_{par}$.

The derivation of the dynamic behaviour of the sensor takes into specific account the application of interest. Let’s consider operating the system with a DC excitation such that $V_{s}$ is constant and assumes a large value, e.g., $V_{s}=20\;V$. In such a case, as already discussed, the linearized small signals sensor behaviour is modelled by the loss parallel resistance $R_{l2}$ and by the parallel capacitance $C_{s2}$. In the presence of pressure pulsations these two components vary, due to the variation of the electron density related to the pressure variations. 

Remembering how these parameters are related to the electron density (Table 1), and considering a harmonic pressure pulsation with small magnitude, they can be written as follows:

(10) $$\frac{1}{R_{l2}}=G_{l2_{0}}\left(1+\alpha \cos\left(\omega t\right)\right)$$

(11) $$C_{s2}=C_{s2_{0}}\left(1+\beta \cos\left(\omega t\right)\right)$$

where *α* and *β* are parameters, and $\alpha =p_{a}R_{l2_{0}}{\left.\frac{\partial G_{l2}}{\partial p}\right|}_{p=p_{0}}$ and $\beta =\frac{p_{a}}{C_{s20}}{\left.\frac{\partial C_{s2}}{\partial p}\right|}_{p=p_{0}}$, with $p_{a}$ indicating the amplitude of the pressure pulsation, $G_{l2}=\frac{1}{R_{l2}}$ indicating the equivalent conductance, and the subscript 0 indicating the average values (DC working point).

As such, defining *v*(*t*) the voltage across the sensors, i.e., the parallel of $R_{l2}$ and $C_{s2}$, and *i*(*t*) the current flowing through the sensor, we can write:

(12) $$i\left(t\right)=G_{l2}\;v\left(t\right)+C_{s2}\frac{dv\left(t\right)}{dt}+v\left(t\right)\frac{dC_{s2}}{dt}$$

Assuming that $v\left(t\right)≅V_{DC}$, i.e., that the pressure pulsations induce small fluctuations of the voltage around its large average value, Equation (12) can be simplified as follows:

(13) $$i\left(t\right)\approx G_{l2}{}_{0}V_{DC}+G_{l2}{}_{0}V_{DC}\alpha \cos\left(\omega t\right)-\omega V_{DC}C_{S2_{0}}\beta \sin\left(\omega t\right)\;=G_{l2}{}_{0}V_{DC}+i_{0}\cos\left(\omega t+\phi \right)$$

Therefore, the sensor behaves as the parallel of $R_{l2_{0}}$, with the addition of a parallel current AC generator. Note that the amplitude of the AC current depends both on the external biasing, on the pressure pulsation magnitude (through the parameters *α* and *β*), and on the frequency. Considering the overall equivalent circuit comprising the excitation voltage source, the parasitic components and the shunt resistor *R_s_*, knowing that *R_s_* ≪ *R*_*l*2_, we find that this equivalent current generator can be replaced by a series AC voltage generators with amplitude approximately equal to *i*_0_*R_s_*, and that the measurement system frequency domain behaviour is a first order low pass filter whose dominant time constant is given by the current sensing resistance *R_s_* and the cable parasitic capacitance $C_{par}$ [10]. Therefore, considering typical values for the parasitic components, for the shunt resistance, and for the sensors parameters, the bandwidth of the measurement system appears practically independent from the working point and from the sensor characteristics. The value of *R_s_* and of *C_par_* must be considered to grant the bandwidth needed for the target dynamical measurements.

## 3. Experimental Setup

### 3.1. Measurement Technique

The ionic current measurement technique used in this work relies on the polarization of the spark plug electrode with a zero-mean low frequency square wave. The selection of an AC excitation ensures the reduction of some problems mainly related to possible chemical or, more properly, electrochemical reactions at the metal/plasma interface that were not considered in the previous treatment but, especially due to the harsh conditions in the combustor, could significantly contribute to the sensor behaviour. For instance, the oxidation of one of the two electrodes is favoured by a constant applied voltage, and this would introduce a large series resistance to the ionic current path. Adopting a low frequency square wave allows for avoiding the electrode polarization related phenomena typical of DC measurements but, on the other hand, requires analysing separately the two portions of the measured current corresponding to the high and the low half period of the excitation signal. Since the focus of the work is the analysis of the combustion chamber pulsations, the ion current signal must be analysed in the frequency domain. For this reason, the square wave frequency has been chosen considering the high and low periods long enough to have a sufficient low frequency resolution when performing the frequency domain signal processing. Moreover, the half periods must be long enough to ensure that the transients of the measurement system triggered by the excitation signal rising and falling edges vanish, and to allow for discarding the portions of the measured signal perturbed by these transient events.

### 3.2. Measurement Setup

**Figure 4:**
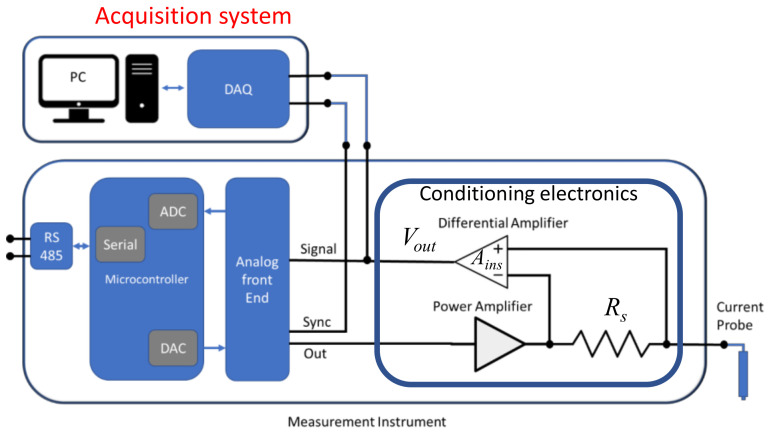
Conditioning electronics and measurement setup.

The ion current was measured using the instrument shown in Figure 4, which generates the excitation signal, and measures the current flowing in the probe. The device is based on a microcontroller (STM32L432) that embeds a 12-bit digital to analogue converter (DAC), used to generate the excitation signal driving the current probe. The signal can be a constant DC, a sinusoidal, a rectangular, or a triangular waveform. The signal shape, together with its amplitude and frequency can be set by a serial interface and a Modbus^®^ protocol. The microcontroller also acquires the signal proportional to the ionic current provided by the conditioning electronics, using a 12-bit analogue to digital converter (ADC). 

Measurements were also performed using an external acquisition board connected to a PC (National Instruments, NI-PCI 6014, 16 bit ADC).

The conditioning electronics consists of a DC coupled low output-impedance amplifier to bias the spark plug electrode, and of a current to voltage converter to perform the current measurement.

The current measurement is performed high side since the spark plug case must necessarily be grounded, it being in electrical contact (due to the mounting) with the gas combustion chamber enclosure. In the spark plug only one floating electrode is available, i.e., the tip.

With reference to Figure 4, the power amplifier can supply a maximum excitation voltage of 55 V_pp_ (peak-peak), and is powered by a dual power supply of ±30 V. The design is based on the OPA454 operational amplifier paired with a push-pull output stage to reduce the output impedance and to ensure a safety margin of the current supply during the rising and falling edges of the square wave.

The current-to-voltage converter consists of the shunt resistance $R_{s}$ and of an instrumentation amplifier, with gain *A_ins_*, realized using, in the input stage, two OPA454 high voltage operational amplifiers. The use of two high voltage operational amplifier is needed due to the peak voltages required by the application.

The measured current is related to the output voltage as follows:

(14) $$I_{Meas}=\frac{V_{Out}}{A_{ins}R_{s}}$$

Considering that the measured current is in the order of several μA, the resistance $R_{s}$ and the gain *A_ins_* were set to 1.5 kΩ and 100, respectively, with a resulting resolution of 150 mV/μA. Moreover, the shunt resistance was selected also considering the maximum bandwidth needed for the dynamic measurements, as discussed in the previous section.

The conditioning electronics were used in different laboratory experiments in conjunction with a LabVIEW virtual instrument executed by a PC controlling the overall measurement set-up, and with a NI DAQ board acquiring the measured current signal (sampling frequency 20 kHz). The output signal of the current to voltage converter (*V_out_* in Figure 4) was processed by analysing separately the high and the low half periods of the square wave and eliminating the first part of each half period affected by the transients. 

In the next section the *V_out_* mean and rms values (high and low half periods) obtained in the different experiments by averaging four subsequent periods using excitation signals with frequency in the range 1–4 Hz and amplitude in the range 0.5–55 Vpp will be presented and discussed.

For the lab experiments, a propane-butane nozzle burner torch with fixed air-fuel premix was used to set a reference flame with fixed combustion conditions. 

**Figure 5:**
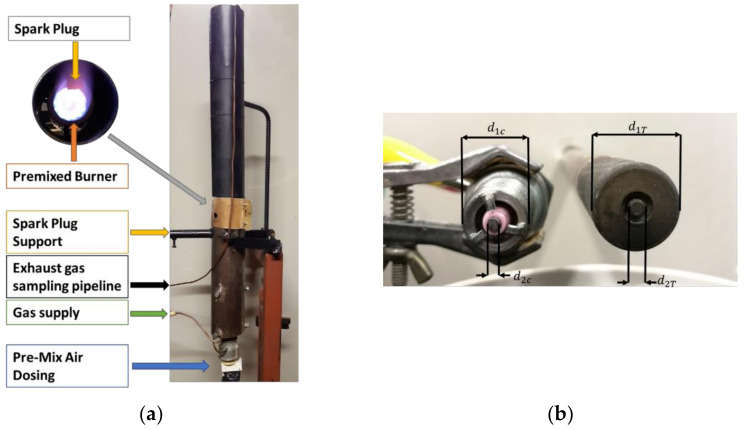
(**a**) The burner test bench used for the experiments. (**b**) The two spark plugs used for the experiments.

In addition, a burner test bench (see Figure 5a) was used to test the flame ion current in different combustion conditions. In this case, the combustible mixtures with a defined air to fuel ratio are obtained exploiting two controlled flows of air and propane (C_3_H_8_). The mixture flow passes through a nozzle that impresses a rotation to the fluid to increase the flame stability. The combustion occurs after this nozzle, and the distance between the nozzle and the current probe (d) can be modified. To keep under control the temperature and avoid the overheating of the mechanical structure of the burner, a flow of cooling air around the burner was used. The probe is positioned before the mixing point of the cooling air to measure only the premixed mixture contribution to the combustion. 

Two different spark plugs, shown in Figure 5b were used in the experiments, an automotive spark (leftmost), with $d_{1c}=12\;\mathrm{mm}$ and $d_{2c}=1.9\;\mathrm{mm}$ and a gap between the tip and the housing of 1.5 mm, and a gas turbine spark plug (rightmost) with $d_{1T}=16\;\mathrm{mm}$, $d_{2T}=3\;\mathrm{mm}$ and a gap between the two electrodes of 1.2 mm. 

**Figure 6:**
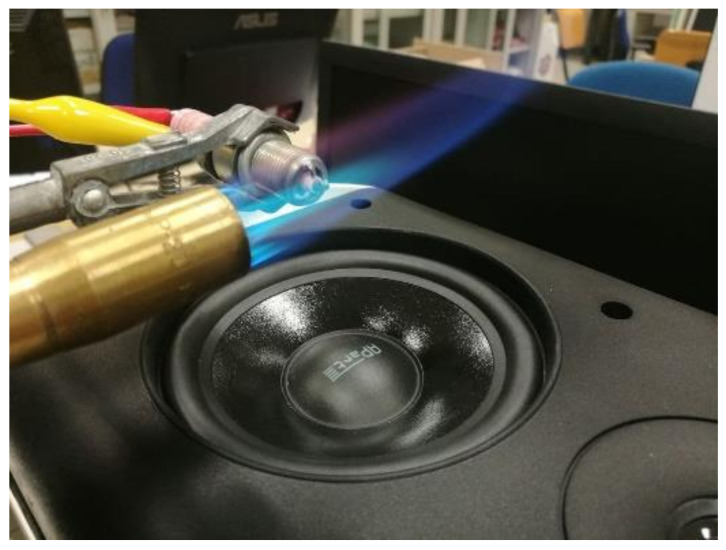
Setup for the acoustic pulsation emulation.

During the lab tests, the pressure pulsations in the combustion chamber were emulated by generating pressure waves using a loudspeaker mounted near the flame. The chosen loudspeaker box has an acoustic suspension design, and we can consider the loudspeaker acting as a piston in a frequency interval from zero to an upper value determined by the cone dimension. [28]. The adopted loudspeaker has a diameter of 16 cm, and can act as a piston in the frequency range 50–150 Hz. As shown in Figure 6, the loudspeaker cone was placed under the flame at a distance of about 5 cm to avoid overheating.

The loudspeaker was driven by a power amplifier and a waveform generator (Agilent, AG 333220A), using sinusoidal signals with frequencies in the range 50–400 Hz. To estimate the amplitude of the pressure variations, $p_{EXC}$, the value of the acoustic pressure was measured by a sound level meter.

## 4. Experimental Results

### 4.1. Laboratory Tests

The gas turbine spark plug was used to experimentally verify the results presented in Section 2 concerning the model of the ion sensor. Before performing the measurements in the presence of combustion, the spark plug was characterized in the absence of flame to obtain an estimation of the parasitic components: in particular, *R_par_* was estimated equal to about 1.95 GΩ.

**Figure 7:**
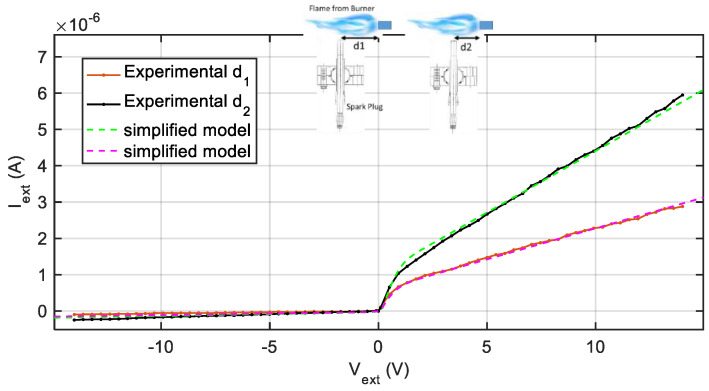
Experimental results (lines with markers) obtained placing the sensor (gas turbine spark plug) at two different distances from the torch nozzle ($d_{1}$ = 6 cm, $d_{2}$ = 3 cm—picture right side). Dashed lines: model linearized at *V_W_* = 5 V.

Figure 7 reports the experimental results obtained by placing the sensor (gas turbine spark plug) at two different distances from the torch nozzle ($d_{1}$ = 6 cm, $d_{2}$ = 3 cm). The model parameters obtained by fitting are: (*d*_1_) *T* = 1273 K, *A*_2_ = 39.5 *A*_1_, *n_s_* = 5.1 × 10^12^ m^−3^, *μ_e_* = 800 m^2^ V^−1^ s^−1^; (*d*_2_) *T* = 2373 K, *A*_2_ = 39.5 *A*_1_, *n_s_* = 1.2 × 10^13^ m^−3^, *μ_e_* = 800 m^2^ V^−1^ s^−1^. The lines with markers represent the mean value of the measured current over 1 s windows as a function of the applied external voltage. The current predicted by the simplified model, presented in Section 2, obtained by non-linear fitting, is superimposed to the experimental data, with the current *I*_*l*2_ obtained as $I_{l2}=\frac{V_{2}}{R_{l2}}$, being *R*_*l*2_ a constant value attained with *V_W_* = 5 V. The estimated ratio between the two electrode areas is 39.5. As expected, changing the spark plug position leads to the estimation of different temperatures and carrier densities. The simplified model describes very well the ion sensor behaviour. This can be due to the contribution of effects that were neglected in the model development, such as, for instance, collisions in the sheath, which cause the relationship of the ion current with the applied voltage to be better described by a linear function than by the square root foreseen by the model.

**Figure 8:**
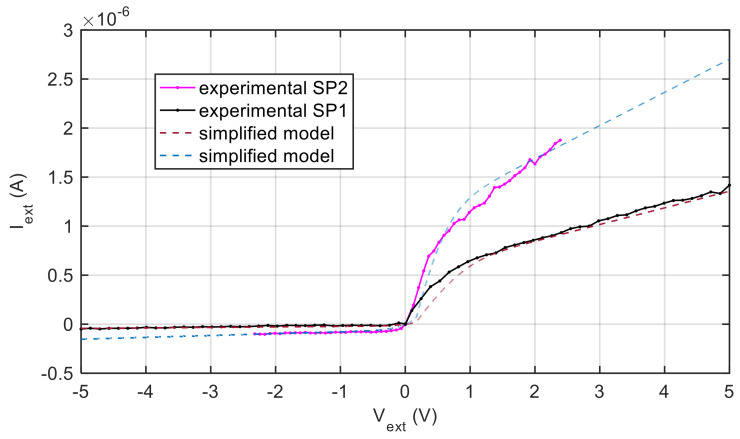
Experimental results (lines with markers) obtained using two different spark plugs (SP1—turbine spark plug and SP2—automotive spark plug), placed at 6 cm from the torch nozzle. Dashed lines: model linearized at *V_W_* = 5 V.

A further example in this sense, reported in Figure 8, is found by comparing the results obtained with the two different spark plugs described in the previous section, placed at 6 cm from the torch nozzle. For the automotive spark plug the estimated ratio between the two electrode areas is 20. The model parameters obtained by fitting are: SP1) *T* = 1273 K, *A*_2_ = 39.5 *A*_1_, *n_s_* = 8.6 × 10^12^ m^−3^, *μ_e_* = 800 m^2^ V^−1^ s^−1^; SP2) *T* = 1273 K, *A*_2_ = 20 *A*_1_, *n_s_* = 3.43 × 10^13^ m^−3^, *μ_e_* = 800 m^2^ V^−1^ s^−1^.

**Figure 9:**
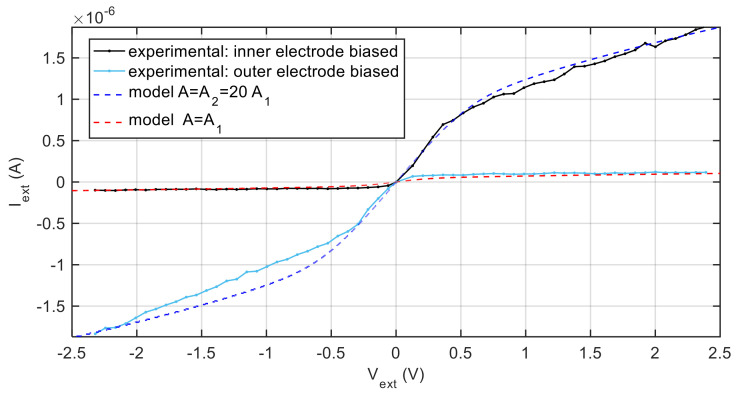
I-V characteristics obtained with the automotive spark plug driving the inner electrode (black solid line with markers), and the outer electrode (cyan solid line with markers). Blue and red dashed lines: model predictions obtained considering two symmetrical sensors, as per legend.

In Figure 9, two I-V characteristics obtained with the automotive spark plug are shown, the first one is derived driving the inner electrodes, whereas the second is obtained driving the outer electrode. The experimental data are compared to the model predictions obtained considering two symmetrical sensors, one with both electrode areas equal to the inner electrode one, the other with a sensor with both electrode areas equal to 20 times the inner electrode areas.

**Figure 10:**
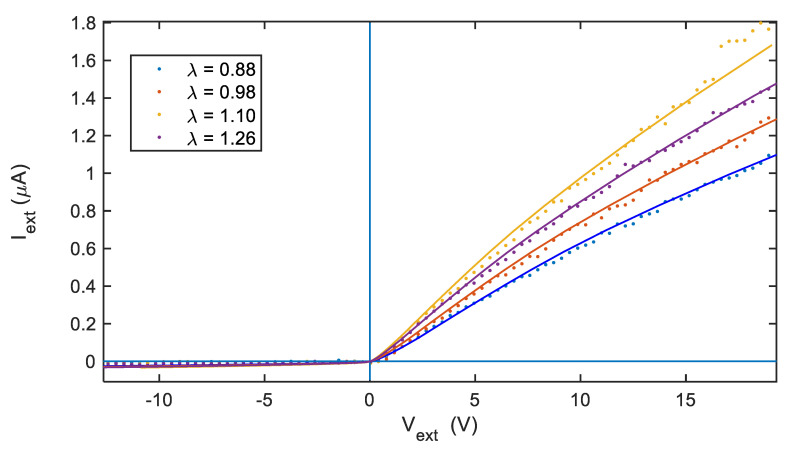
I-V characteristics obtained with the turbine spark plug and the laboratory test burner for different air to fuel equivalent ratios λ, as per legend. Dots: experimental data; solid lines: model fitting.

Finally, tests in the laboratory burner (reported in Figure 10) show the case of very large ratios between the two electrode areas (*A*_2_ = 39.5 *A*_1_) and also how the I-V characteristic is influenced by the combustion conditions, which is explained through model fitting by different ion concentrations and flame temperatures as reported in Table 2.

Note that for the measured current values reported in the presented I-V curves the uncertainty affecting the experimental data was mainly attributed to the turbulent nature of the observed phenomenon. Data were gathered while the combustion was nominally at a regime so that for each sample of the I-V characteristics, evaluating the average over longer windows using longer measurement times should have reduced the uncertainty. Nevertheless, a too long measurement time implies a very long time for the whole experiment, in which environmental changes could perturb the combustion regime itself. Therefore, the measurement time was selected as a tradeoff. The variability estimated in the adopted working conditions for the I-V characteristic assessment leads to an uncertainty lower than 10% of the measured values in all the tests.

Dynamic measurements were performed in the laboratory exploiting the measurement set up described in the previous section (Figure 6) and the gas turbine spark plug. It must be highlighted that, considering the estimated parasitic components for this spark plug and the front-end circuit parameters values, the expected bandwidth of the measurement system is about 10 kHz. The measurements were performed using single tones with frequency in the interval 50–400 Hz and acoustic pressure amplitudes in the range 0.5–130 Pa. The pressure pulsation amplitudes used in these tests are similar or smaller than those present in a real combustor chamber. However, differently from the laboratory test conditions, in the combustion chamber there is an average pressure larger than the atmospheric pressure, given by the compressor and the gas turbine operating conditions, which in absence of pulsations is almost constant. 

**Figure 11:**
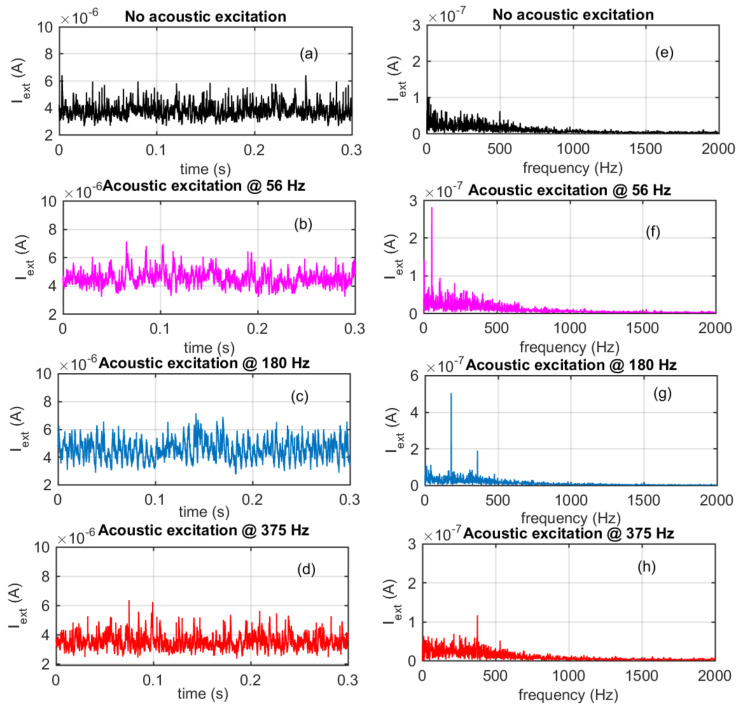
Signals from the spark plug front end circuit in the absence and in the presence of acoustic excitation as a function of time (**a**–**d**), and FFT of the acquired signals (**e**–**h**).

Figure 11a,e show the typical behaviour of the ion current in the absence of acoustic excitation, which has a mean value related to the average combustion conditions (time averaged fluid dynamical condition) with superimposed random and large-bandwidth fluctuations related to the fluid dynamical turbulence intrinsic in the observed combustion process. The statistical characteristics (probability density function) of these fluctuations are related to the combustion conditions.

Figure 11b–d display the acquired signals vs. time and Figure 11f–h the corresponding FFTs, in the presence of acoustic excitations. In detail, Figure 11b,f show the effect of an acoustic excitation at 56 Hz with an amplitude of $107\;{\mathrm{dB}}_{\mathrm{SPL}}$, corresponding to 5 Pa; whereas Figure 11c,g present the effect of an acoustic excitation at 180 Hz, with an amplitude of $136\;{\mathrm{dB}}_{\mathrm{SPL}}$, corresponding to 130 Pa; Figure 11d,h show the effect of an acoustic excitation at 375 Hz, with amplitude of $128\;{\mathrm{dB}}_{\mathrm{SPL}}$, corresponding to 55 Pa. In the presence of the induced pressure pulsations the signal spectra clearly show a peak at the excitation frequency with an amplitude much larger than the noise floor; in fact, even in case of the smallest pressure amplitude, the peak amplitude is about three times the noise floor related to the turbulence signal.

**Figure 12:**
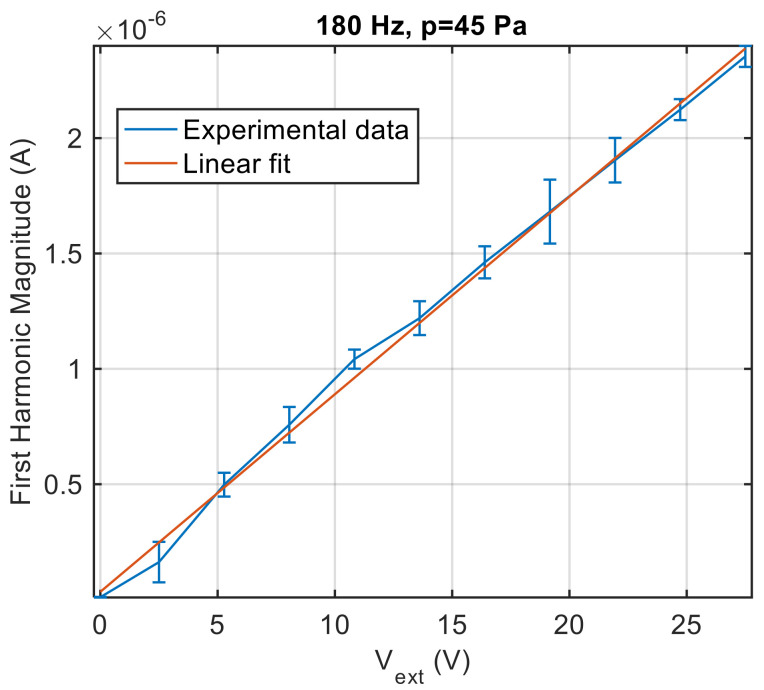
Peak magnitude of the ion current first harmonic as a function of the spark plug excitation voltage (peak-to-peak amplitude) with an acoustic pressure of 120 Pa at 180 Hz. Measured current values are the mean values obtained from seven repeated experiments, error bars represent the standard deviations.

To validate the study on the dynamical behaviour of the ion sensor reported in Equation (13), measurements to confirm the linear dependence of the sensor response magnitude on the spark plug excitation voltage and on the pressure wave amplitude were performed. Figure 12 reports the first harmonic magnitude as a function of the spark plug excitation voltage amplitude obtained in the presence of an acoustic pressure wave with amplitude of 120 Pa at 180 Hz, i.e., near the resonance peak of the loudspeaker enclosure, where low distortion is expected. The measurements were repeated seven times, and the figure shows the average value and the standard deviation obtained from repeated measurements (error-bar). As it can be seen, the amplitude of the first harmonic increases linearly with the excitation voltage. Considering, also in this case, the measurement type A uncertainty, mainly due to the turbulence of the observed process, as the main source of uncertainty, it can be seen how the presence of pressure pulsations with moderate amplitude (120 Pa) can be observed even exploiting very low excitation voltages (down to a few volts).

**Figure 13:**
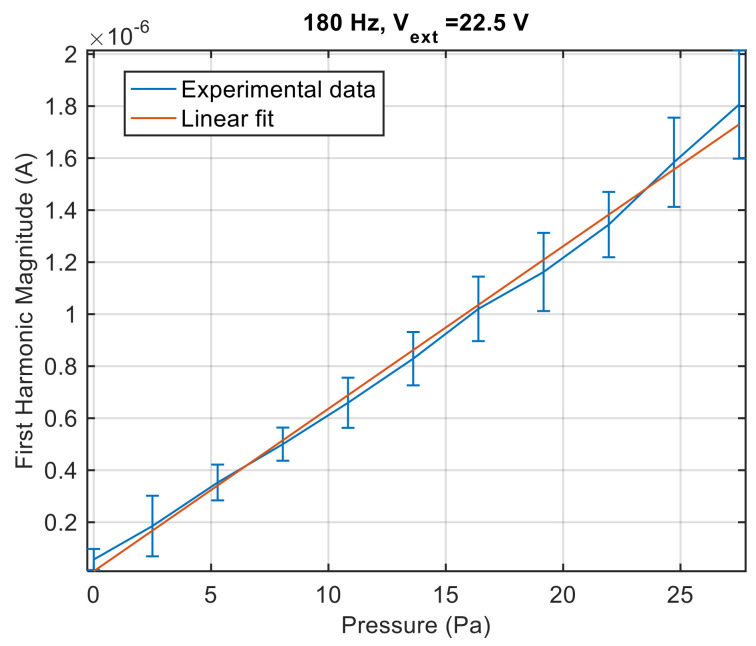
Peak magnitude of the ion current first harmonic as a function of the acoustic pressure at 180 Hz. V_ext_ = 22.5 V. Measured current values are the mean values obtained from 10 repeated experiments, error bars represent the standard deviations.

Figure 13 shows instead the first harmonic magnitude as a function of the acoustic pressure at a fixed excitation voltage V_ext_ = 22.5 V. Also, in this case the experimental results confirm the proposed model, and in these conditions the proposed system allows for detecting small amplitude pressures (down to about 10 Pa).

### 4.2. In Field Measurements

The measurement system based on the ion current sensor was tested also in field, at an atmospheric test rig located in the Nuovo Pignone (Baker Hughes) facility in Florence. The test rig consists of a combustion chamber able of emulating the typical gas turbine operating conditions. This combustion chamber is fully instrumented and provided with many different sensors, including two piezoelectric dynamic pressure sensors. The test rig is controlled and monitored by a remote system, able to acquire, process, save and display in real time both control signals (e.g., fuel and air mass flows controls) and signal from the on board sensors. 

The combustor also hosts as a spare spark plug with the geometry taken into account in this work and used for the laboratory tests presented and discussed in the previous sub-section. 

For the in field tests, the spare spark plug was connected to the stand-alone instrument described in Section 4, and was able to acquire and save pre-processed data locally with the aid of a PC. Moreover, the output of the conditioning electronics (ion current analogue signal coming from the current-to-voltage converter) was connected to the test rig control and monitoring system, which can acquire the signal with a high sampling frequency (all the signals are low-pass filtered and then A/D converted at 10 kHz and 16 bits), save the high frequency data stream (together with all the other relevant control and sensors signals) in the experiment server memory, and display it in real time during tests.

The test rig combustion chamber can work both with methane and hydrogen fuel, and the operating conditions are set by controlling the air and the fuel flows in terms temperature and speed. The burner can be fed with different air/fuel ratios and different fuels (also mixing methane and hydrogen), adjusting the combustion chamber power.

As already said, the combustion chamber is instrumented with the equipment usually present for condition monitoring of real turbomachines.

**Figure 14:**
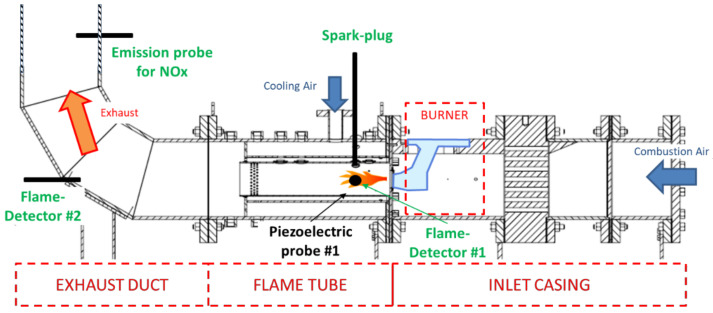
Test rig configuration.

In Figure 14 the test rig configuration is reported. Near the spark plug used for the measurement, the presence of a piezoelectric dynamic pressure probe (labelled Piezoelectric Probe #1) is highlighted. This was used as a reference to validate the capability of the proposed ion current sensor of sensing the pressure variations and pulsation in the combustion chamber. 

In the combustion chamber we can have two types of relevant events related to fast pressure variations; in fact, besides acoustic pulsations given by resonances of the combustion chamber, pressure spikes caused by abrupt working condition variations can also occur. The first type of acoustic pulsations are quasi-harmonic pressure fluctuations and can be associated with the presence of peaks at certain typical frequencies in the FFT of the ion current signal, whereas the second ones are broadband impulsive events associated with large magnitudes and fast variations of the ion current signal, which usually do not excite resonance phenomena inside the chamber. 

For the in-field tests, the spark plug was excited with a square wave with a frequency equal to 4 Hz and an amplitude of $50{\;V}_{\mathrm{pp}}$. The frequency was also selected in order to facilitate the integration of the ion sensor with the control and monitoring system of the test rig.

The results concerning the ‘in field’ measurements reported in this subsection exploit both the static and dynamic response of the ion current sensor, given by variations in the combustion chamber operating conditions. 

In particular, the experimental results reported in this section are obtained during a test in which the combustor started burning methane and then gradually passed to hydrogen combustion by mixing the two fuels during the transition. The test lasted three hours.

Note that this test is particularly challenging for the proposed monitoring technique which, until now, has proven its effectiveness for hydrocarbon combustion only. In fact, in the literature the ion sensing monitoring is linked to the study of the chemo-ionization processes involved in the burning of hydrocarbons. Therefore, the results reported in this section are novel and show that even with a reduced sensitivity the proposed technique is applicable also to the monitoring of combustions with alternative low-carbon fuels.

During the hydrogen combustion, some induced-flashback tests were performed. Flashback (FB) can induce ignition in the premixing zone, which is an imminent risk typical of gas turbine swirl combustors in lean premixed operations and can lead to damage and failure of components. Thus, steady combustion in the premixing zone must be avoided under all circumstances.

During the induced-flashback test, the combustion is forced to occur in the premixer, between the swirler present in the first section of the burner and the burner exit section. During these events, the ion current signal magnitude is very low, since the spark plug is placed in a region of the combustor very far from the flame. Usually, at the beginning of the induced-flashback event, a pressure peak given by the premix zone ignition occurs.

After the induced-flashback event, a re-ignition of the flame in the normal location occurs; this usually causes another large pressure pulse in the combustor. After re-ignition the flame is present in the combustion chamber, therefore the ion current signal can sense the pressure variation and, if present, the pressure pulsations. 

To validate the results obtained from the analysis of the acquired ion current signal, the output of a dynamic pressure sensor (piezoelectric) was used as a reference. This sensor was placed in the combustor, at the same axial distance from the nozzle as the spark plug. Since the test took three hours because of the large amount of data, time sequences consisting of the RMS values of the ion current signal and of the pressure signal, evaluated over time windows of 1 s, were analysed first.

Peaks of the amplitudes of these time sequences were interpreted as relevant events, either related to induced-flashback tests or to possible pressure oscillations. The detected event times were then checked using the experiment time-schedule. After this first analysis, which allowed for identifying the ‘relevant events’, the corresponding portions of the two signals, sampled at high frequency, were analysed in the time and frequency domains.

**Figure 15:**
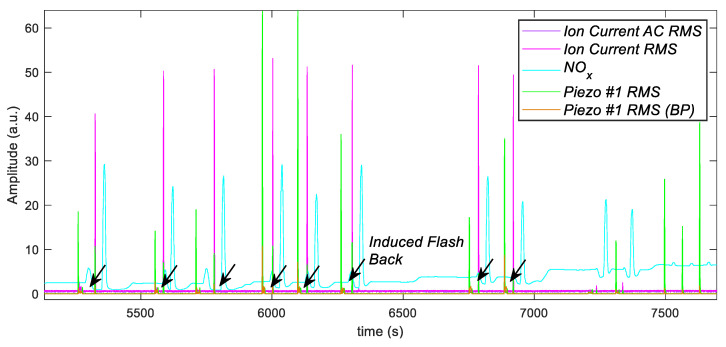
Time sequences of the measured parameters during the test. Piezo #1 RMS and Piezo #1 RMS (BP): scaled pressure measurements from the sensor placed inside the combustor, RMS value and RMS value in a narrower bandwidth, respectively; Ion Current RMS and Ion Current AC RMS: measured ion current expressed in RMS and RMS of the AC component, respectively; NOx: NOx normalized concentration (a.u.).

Figure 15 shows the time sequences of some selected measured parameters provided by the test rig instrumentation (time step 1 s). These parameters were scaled and represented in arbitrary units to allow a simple interpretation of the data.

The selected parameters comprise the dynamic pressure measurements provided by the piezoelectric pressure sensor mounted on the test rig (Figure 14). The measured parameters are the pressure RMS values in the whole measurement frequency band (0 Hz–5 kHz), whereas piezo #1 BP indicates the RMS value in a narrower band (500 Hz–2000 Hz). The plot reports also the normalized measured NO_x_ concentration, which can be used as an index of the combustion quality, as well as the measured ion current, expressed here in terms of RMS (Ion Current RMS) and standard deviation, i.e., RMS of the AC component (Ion Current AC RMS). 

As expected, there is a clear correlation between the ion current measurement, which presents large peaks in correspondence to induced-flashback events (highlighted with arrows in Figure 15) and pressure measurements, but also with NO_x_ emission measurements. 

Combustion chamber acoustic pulsations mostly occur during ignitions and high-power demanding conditions. During the test, the combustion chamber has been driven decreasing the power gradually over time during the passage from methane to hydrogen fuel. For this reason, acoustic pulsations occurred only during the first phases of the test, when the combustor burned methane fuel.

In particular, the spectra of the pressure and ion current corresponding to an event of pressure oscillations are shown in the following figures. The ion current signal was processed by averaging the FFT of the high signal half periods over a time interval of 30 s. The pressure signal was analysed using the same time windows and in the same way. 

**Figure 16:**
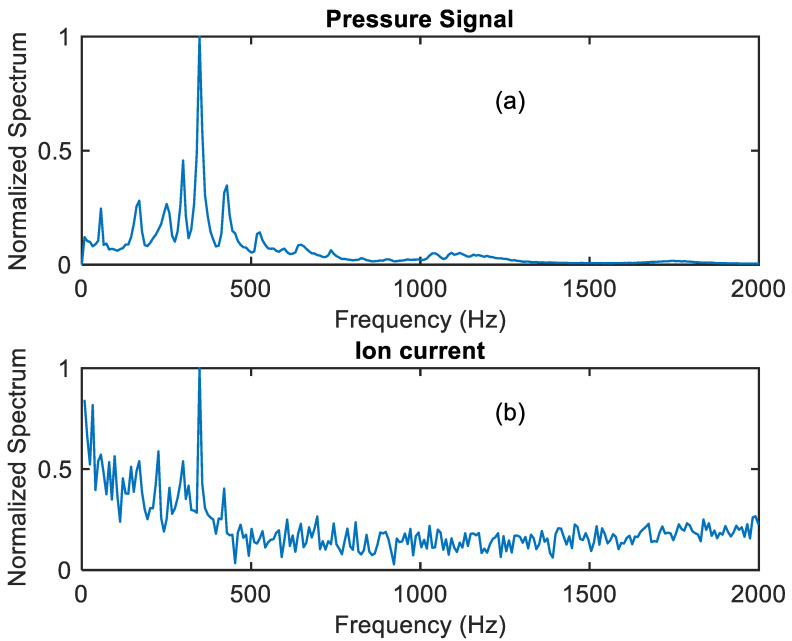
Spectra of the pressure (plot (**a**)) and of the ionic current (plot (**b**)) signals during a combustion chamber pulsation event at 348 Hz (arbitrary units).

Figure 16 shows the spectra of the pressure and of the ionic current signals during a combustion chamber pulsation event at 348 Hz, both expressed in arbitrary units, showing the capability of the proposed system of capturing the pressure oscillations in real applications.

## 5. Conclusions

This paper demonstrated the possibility of using a sensing strategy based on the exploitation of a rugged component, the spark plug (present in the combustion chamber of gas turbines) to obtain an effective monitoring of the combustion. In particular, the possibility of detecting pressure fluctuations in the combustion chamber was proven. This is particularly important because the detection of pressure oscillation or fluctuation can be used for early detection of lean blowout (LBO) or other combustion instability conditions. It was shown how the spark-plug works as an ion density sensor, through a model based on the Langmuir probe theory, that is able to account both for the static and dynamic sensing behavior. The relationship of the sensor response to the electrodes areas was elucidated. The developed model is verified through laboratory experiments in stationary and non-stationary conditions, i.e., in the presence of induced small pressure fluctuations. The results obtained showed an acceptable uncertainty (below 10% of the measured current amplitude) which is mainly related to the instability and turbulence of the observed phenomenon and demonstrated an achievable resolution of about 2 Pa on the estimated pulsation amplitude, even under small biasing voltage (22.5 V), with an estimated bandwidth of 10 kHz. The main advantages of using the proposed measurement system are mainly related to its robustness, simplicity, and large bandwidth, Moreover, since it was positioned in the combustion chamber it could give ‘direct’ information on the combustion. 

Finally, the in-field test confirmed the applicability of the proposed sensing system, showing a strong correlation between the combustion monitoring results obtained with the ion sensor and those given by conventional dynamic pressure sensors.

## Figures and Tables

**Table 1:** Complete equivalent circuit parameter definition.

	Parallel Capacitance	Diodes 1 and 2	Constant Current Generators 1 and 2	Loss Resistances	Gas Path Resistance
${\left(V_{p}-\;V\right)}_{j}=V_{j}>0$ *j* = 1, 2	$C_{S_{j}}=\frac{\epsilon \;A_{j}}{\lambda_{D}}$	$I_{D_{j}}=I_{Sj}\left(e^{-\frac{V_{j}}{V_{T_{j}}}}-1\right)$; $I_{Sj}=en_{s}A_{j}v_{e}^{\prime }$; $V_{Tj}=\frac{kT_{e}}{e}$	$I_{ion_{j}}=en_{s}A_{i}\left(v_{bj}^{\prime \prime }-v_{ej}^{\prime }\right)$	$R_{l_{j}}={\left|\frac{dI_{si_{j}}}{dV_{j}}\right|}^{-1}$	$R_{p}=\frac{0.61L}{n_{s}A_{1}e\;\mu_{e}}$ If $A_{1}<A_{2}$
${\left(V_{p}-\;V\right)}_{j}=V_{j}<0$ *j* = 1, 2		$I_{D_{j}}=I_{Sj}\left(e^{\frac{V_{j}}{V_{T_{j}}}}-1\right)$; $I_{Sj}=-en_{s}A_{j}v_{b}^{\prime }$; $V_{Tj}=\frac{kT_{e}}{e}$	$I_{ion_{j}}=-en_{s}A_{i}\left(v_{e_{j}}^{\prime }-v_{bj}^{\prime \prime }\right)$	$R_{l_{j}}={\left|\frac{dI_{se_{j}}}{dV_{j}}\right|}^{-1}$	

**Table 2:** Assessed model parameters for data reported in Figure 10.

Air-Fuel Equivalence Ratio (λ)	*n_s_* (m^−3^)	*μ_e_* (m^2^/ (V s))	*T_e_* (K)
0.88	1.15 × 10^13^	80	2000
0.98	1.35 × 10^13^	80	1500
1.10	1.78 × 10^13^	80	1000
1.26	1.55 × 10^13^	80	1000

**Figure A1:**
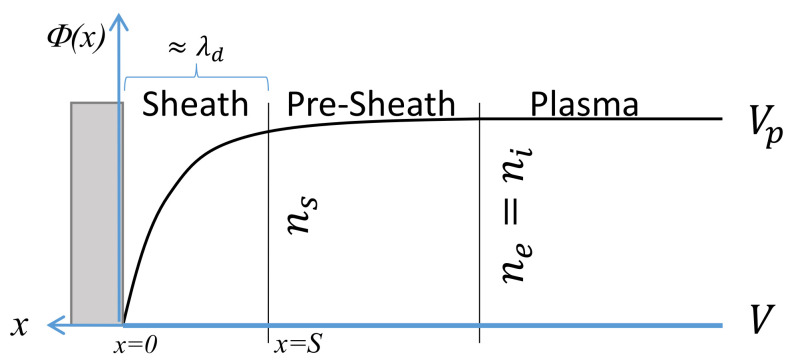
Voltage close to the plasma—object interface vs. distance.

## Appendix A. Derivation of Equation (1)

In the combustion chamber, the free charges (ions and electrons) are generated by the combustion reactions. Neglecting the fluctuation due to turbulence and considering only the average values, when the reactions are at the steady state the ion and electron density average can be considered constant, and we have:

(A1) $$\frac{\partial n_{e}}{\partial t}=\frac{\partial n_{i}}{\partial t}=0$$

where *n_e_* and *n_i_* are the ion and electron density average values. In this situation the current flowing through the electrodes can be determined knowing the density of charges determined by the equilibrium constant of the reactions that cause the ionization.

The ionized gas in the combustion chamber can be treated as a plasma, in fact the flame temperature can range from 600 K in the outer regions to 5000 K [24] and the electron density is typically in the order of $10^{6}\frac{1}{{\mathrm{cm}}^{3}}$–10^8^ $\frac{1}{{\mathrm{cm}}^{3}}$ [21], even in the region close to the spark plug where the temperature is typically about 600 K. Therefore, from now on we will model the flame as a plasma.

In such conditions, the velocity of the electrons, $v_{e}$, can be modeled by a Maxwellian distribution:

(A2) $$f\left(v_{e}\right)={\left(\frac{m_{e}}{2\pi kT_{e}}\right)}^{\frac{1}{2}}e^{\left(-\frac{m_{e}v_{e}^{2}}{2kT_{e}}\right)}$$

where *k* is the Boltzmann constant, *v_e_* is the electron speed along one direction, *m_e_* is the electron mass, whereas *T_e_* is the electron temperature. A similar expression is valid for ions.

Note that, considering that the typical combustion chamber pressures are larger than or equal to the atmospheric pressure, the electron temperature is almost equal to the ion temperature and finally also to the gas temperature, i.e., $T_{e}\approx T_{i}$ and the distribution of the species in the plasma can be described by equilibrium relationships [25]. The plasma by itself can be considered highly conductive and equipotential at a value $V_{p}$ and the space region occupied by the plasma can be considered quasi neutral, i.e., $n_{e}=n_{i}$.

In this appendix the expression for the transport current flowing through an electrode/plasma interface will be derived, accounting for the physical (not chemical) interaction between the plasma and the electrodes [22,23].

To this end we consider at first the space region close to the interface between the electrode and the plasma, the so-called sheath [29], and we consider a 1D geometry, where the surface of the electrode is approximated with a plane infinite surface.

It is well known that at the interface with a material surface, the accumulation of negative charge in the object, and of the positive charge around it, generates a potential barrier for electrons called a Debye sheath. Ions can travel through the sheath, experiencing a small acceleration, but it can be proven, by analysing the electrical field in the sheath, that only those ions that enter in the sheath with a velocity greater than the so-called Bohm velocity, $v_{b}$, will reach the object surface, where:

(A3) $$v_{b}=\sqrt{\frac{kT_{e}}{m_{i}}}$$

where $m_{i}$ is the ion mass. The current which flows from the plasma to the object is composed by the superimposition of the electron current hindered by the electric field in the sheath and of the current due to positive ions. We refer to the 1D problem as described in Figure A1, where it can be seen the existence of a pre-sheath region is assumed, where a small electric field exists.

Considering the potential $\Phi \left(x\right)$ at a generic point *x*, and assuming the object at a potential lower than the one of the plasma, we have $\Phi \left(S\right)>\Phi \left(0\right)$, and only the electrons with a sufficient kinetic energy can cross the sheath region. The energy conservation for these particles is:

(A4) $$\frac{1}{2}m_{e}v_{e}^{2}\left(S\right)-\frac{1}{2}m_{e}v_{e}^{2}\left(0\right)=e\left|\Phi \left(S\right)-\Phi \left(0\right)\right|$$

From the energy conservation it can be found that the minimum speed at the interface at *x* = *S*, of those electrons overcoming the barrier is:

(A5) $$v_{e_{\mathrm{MIN}}}\left(S\right)=\sqrt{\frac{2\left|\Phi \left(S\right)-\Phi \left(0\right)\right|}{m_{e}}}$$

Differently form the bulk plasma, which is neutral (and $n_{i}=n_{e}=n_{b}$), in the sheath $n_{i}\neq n_{e}$, and both $n_{i}=n_{i}\left(x\right)$ and $n_{e}=n_{e}\left(x\right)$ are not uniform along the x axis. Nevertheless, the charge continuity imposes that the electron current density, $J_{e}$, is conserved along the sheath, hence:

(A6) $$J_{e}=-e\;n_{ev}\bar{v_{e}\left(S\right)}=-e\;n_{e}\left(0\right)\bar{v_{e}\left(0\right)}\;$$

where $n_{ev}$ is the density of elctrons with speed larger than the minimum speed of Equation (5) at *x* = *S*, whereas $\bar{v_{e}\left(S\right)}$ is the average speed of electrons with $v_{e}\left(S\right)>v_{e_{\mathrm{MIN}}}\left(S\right)$, and $\bar{v_{e}\left(0\right)}$ is the average speed at the object surface. Therefore, considering the Maxwellian distribution in Equation (3), we can calculate the electron current density as follows:

(A7) $$J_{e}=E[-e\;n_{e}\left(S\right)v_{e}\left(S\right)|v_{e}\left(S\right)\;>v_{e\mathrm{MIN}}\left(S\right)]=-e\;n_{s}\int_{v_{e}{}_{\mathrm{MIN}}\left(S\right)}^{\infty }{\left(\frac{m_{e}}{2\pi kT_{e}}\right)}^{\frac{1}{2}}e^{\left(-\frac{m_{e}u^{2}}{2kT_{e}}\right)}u\;du=-e\;n_{s}v_{e}^{\prime }e^{\frac{-e\;\left|\Phi \left(S\right)-\Phi \left(0\right)\right|}{kT_{e}}}$$

where and $x\in \left[0,S\right]$, $n_{s}=n_{e}\left(x=S\right)=n_{b}e^{-\frac{eV_{pre}}{kT_{e}}}=n_{b}e^{-\frac{1}{2}}≅0.61n_{b},$ being $V_{pre}=\frac{kT_{e}}{2e}$ the pre-sheath voltage drop, and finally $v_{e}^{\prime }=\sqrt{\frac{kT_{e}}{2\pi m_{e}}}$.

The ion current is usually derived considering all ions accelerated to the Bohm velocity in the presheath, therefore we can write:

(A8) $$J_{i}=en_{b}v_{b}=en_{s}v_{b}^{\prime }$$

where $v_{b}^{\prime }=v_{b}e^{\frac{1}{2}}$.

The ion current is usually considered independent from the applied voltage, since the effect of the voltage on velocity is considered counterbalanced by the variation of the sheath thickness, therefore it is assumed that it depends only on the bulk ion density and on the Bohm velocity. Some other effects, which can contribute to the overall current in the sheath around a solid object floating in a plasma, could be taken into account, such as a secondary electron emission from the surface caused by the plasma electron impacts and, more importantly, collisions in the sheath. In any case, considering also these aspects gives results quite similar to those reported in the first part of this appendix [30], leading to the same functional relationships among the physical quantities of interest. Therefore, since this analysis has the final aim of deriving a model for the ion current measurement system, the effect of secondary electron emission and of collisionality will not be considered in the following. A last comment concerns the choice of performing a 1D analysis, which doesn’t consider the sheath expansion away from a 3D surface, causing the total area of the sheath to increase with the thickness of the sheath, as well as the flow of particles and the collected current. Again, we consider that this effect doesn’t significantly modify the parts of the discussion relevant to model derivation.

Note that when considering a floating object immersed in the plasma, the equilibrium condition is reached when the electric field in the sheath ensures a net current equal to zero; in summary, the equilibrium is reached at a sheath potential equal to *V_f_*, such that:

(A9) $$J_{i}+J_{e}=0$$

which implies:

(A10) $${\left(\Phi \left(S\right)-\;\Phi \left(0\right)\right)}_{eq}=V_{f}=\frac{kT_{e}}{e}\ln\left(\frac{n_{s}}{n_{b}}\sqrt{\frac{m_{i}}{2\pi m_{e}}}\right)$$

Applying a potential to the wall negative with respect to the plasma potential, and different from *V_f_*, leads to a net current flowing in the sheath. In this case, an I-V model for a plane electrode with area *A* biased with a voltage V based on an equivalent junction [31,32], can be derived by writing the total transport current in the sheath, $I_{t}$, exploiting Equations (A7) and (A8):

(A11) $$I_{t}=en_{s}A\left(v_{b}^{\prime }-v_{e}^{\prime }\right)-en_{s}Av_{e}^{\prime }\left(e^{\frac{-e\left(V_{p}-V\right)}{kT_{e}}}-1\right)$$

where we can observe that the current described by Equation (A11) consists of two terms, the leftmost one that can be modeled by an equivalent constant current generator, $I_{ion}$, and the rightmost term, which can be represented by an equivalent diode contributing with a current $I_{D}$, hence:

(A12) $$I_{t}=I_{ion}-I_{D}$$

The current $I_{ion}$ was derived considering a constant ion velocity over the sheath. On the other hand, the accelerating effect of the electric field on ions can be modelled by exploiting the energy conservation in the sheath:

(A13) $$v_{i}{\left(x\right)}^{2}\;=\frac{2e\left(\Phi \left(S\right)-\Phi \left(x\right)\right)}{m_{i}}+v_{b}^{2}$$

where $x\in \left[0,S\right]$, and where $v_{i}\left(x\right)$ and $\phi \left(x\right)$ are the ion velocity and the potential at x, respectively. Therefore, the velocity of the ions $v_{w}$ at the object interface will be:

(A14) $$v_{w}=v_{b}\sqrt{1-\frac{2e}{kT_{e}}\left(V-V_{p}\right)}$$

Because $\Phi \left(S\right)-\Phi \left(0\right)\approx V_{p}-V$.

This leads to modifying Equation (A11) into the following equation:

(A15) $$I_{t}=en_{s\;}Av_{b}^{\prime }\sqrt{1+\frac{2e\left(V_{p}-V\right)}{kT_{e}}}-en_{s}Av_{e}^{\prime }-en_{s}Av_{e}^{\prime }\left(e^{\frac{-e\left(V_{p}-V\right)}{kT_{e}}}-1\right)$$

which is the expression used for the sensor equivalent circuit derivation, and reported in Equation (1).
